# A Novel Classification for Predicting Chronic Total Occlusion Percutaneous Coronary Intervention

**DOI:** 10.3389/fcvm.2022.762351

**Published:** 2022-02-28

**Authors:** Dongfeng Zhang, Haoran Xing, Rui Wang, Jinfan Tian, Zhiguo Ju, Lijun Zhang, Hui Chen, Yi He, Xiantao Song

**Affiliations:** ^1^Department of Cardiology, Beijing Anzhen Hospital, Capital Medical University, Beijing, China; ^2^Department of Radiology, Beijing Friendship Hospital, Capital Medical University, Beijing, China; ^3^College of Medical Imaging, Shanghai University of Medicine & Health Science, Shanghai, China; ^4^Department of Radiology, Beijing Anzhen Hospital, Capital Medical University, Beijing, China; ^5^Department of Cardiology, Beijing Friendship Hospital, Capital Medical University, Beijing, China

**Keywords:** chronic total occlusion, percutaneous coronary intervention, computed tomographic angiography, plaque composition, coronary artery disease

## Abstract

**Aims:**

Chronic total occlusion (CTO) percutaneous coronary intervention (PCI) is characterized by a low success rate and an increase in complications. This study aimed to explore a new and simple classification method based on plaque composition to predict guidewire (GW) crossing within 30 min of CTO lesions.

**Methods:**

This study consecutively enrolled individuals undergoing attempted PCI of CTO who underwent coronary computed tomographic angiography (CCTA) within 2 months. Lesions were divided into soft and hard CTO groups according to the necrotic core proportion.

**Results:**

In this study, 207 lesions were divided into soft (20.3%) and hard CTO (79.7%) groups according to a necrotic core percentage cutoff value of 72.7%. The rate of successful GW crossing within 30 min (57.6 vs. 85.7%, *p* = 0.004) and final success (73.3 vs. 95.2%, *p* = 0.001) were much lower in the hard CTO group. For patients with hard CTO, previous failed attempt, proximal side branch, bending > 45 degrees calcium ≥ 50% cross-sectional area (CSA), and distal reference diameter ≤ 2.5 mm were demonstrated to be associated with GW failure within 30 min. For patients with soft CTO, only blunt entry was proved to be an independent predictive factor of GW failure within 30 min.

**Conclusions:**

Grouping CTO lesions according to the proportion of necrotic core is reasonable and necessary in predicting GW crossing within 30 min. A soft CTO with a necrotic core is more likely to be recanalized compared with a hard CTO with fibrous and/or dense calcium. Different plaque types have variable predictive factors.

## Introduction

A coronary chronic total occlusion (CTO) is generally accepted as 100% occlusion of a coronary artery for a duration ≥ 3 months. Approximately, one-third of coronary artery disease (CAD) is partly due to CTO lesions that are identified by coronary angiography (1). CTO was once treated as the last frontier of interventional cardiology because of its lower success rate and higher risk of complications. Nevertheless, continuous efforts have been made to increase the success rate of CTO percutaneous coronary intervention (PCI) in view of numerous clinical benefits, including reduced angina pectoris, increased ventricular function, and improved quality of life (2, 3).

In the past few decades, the rate of successful CTO PCI has steadily increased due to the development of equipment, progression of technology, and accumulation of operation experience. Meanwhile, several systems were developed to determine the grade of difficulty likely to be encountered in CTO PCI (4–9), using scoring systems helped in selecting the appropriate candidates and optimizing treatment strategies. However, subsequent validation trials have revealed that the predictive values of existing models are unsatisfactory (10). Patients with low scores indicating low difficulty always experience failed procedures and vice versa. We thus assume that some potential factors significantly influence CTO PCI outcomes.

Plaque compositions vary with CAD type and are significantly associated with prognosis (11). CTO pathophysiology revealed that plaque composition with the gradual replacement of cholesterol and foam cells with fibrous and calcification may be an important factor associated with the feasibility of passaging the occlusion with a wire (12). However, previous scoring systems only considered the impact of calcification and disregarded other components. Coronary computed tomographic angiography (CCTA) is a useful diagnostic tool for the analysis of coronary plaques (13). Therefore, this study aimed to explore a new classification method based on CCTA derived plaque composition to predict the guidewire (GW) crossing within 30 min of CTO lesions.

## Materials and Methods

### Study Design and Population

We retrospectively enrolled consecutive patients undergoing attempted PCI of invasive coronary angiography (ICA) confirmed CTO with a CCTA performed within 2 months before ICA in the time period between September 2015 and September 2019 from two high volume centers (Beijing Anzhen Hospital and Beijing Friendship Hospital, Beijing, China). CTO was defined as Thrombolysis in Myocardial Infarction (TIMI) flow grade 0 in a native vessel and estimated to have lasted for at least 3 months according to the first onset of angina pectoris, previous history of myocardial infarction, or comparison with a prior angiogram. All enrolled candidates had either typical angina symptoms or functional tests that demonstrated myocardial ischemia.

The percutaneous coronary intervention was performed by experienced interventional cardiologists with a minimum of 50 CTO cases per year. The interventional strategies, including GW selection and crossing approach, were left to the discretion of the operator. Except for CTO vessels that were supplied only by ipsilateral collaterals, the use of bilateral injection was essential. Crossing wires were selected in a step-up approach beginning with soft polymeric wires and then stiff flat or tapered wires. Antegrade approaches including the manipulation of stiff wires and parallel wire technique as well as retrograde approach were used.

The primary endpoint was a successful GW crossing through the CTO lesion within 30 min as described in the J-CTO trial (4). Furthermore, GW crossing at any time was set as a secondary outcome. The study protocol was approved by the Institutional Review Boards of Beijing Anzhen Hospital (2020070X) and Beijing Friendship Hospital (2020-P2-228-01) and was conducted according to the principles of the Declaration of Helsinki.

### CCTA Protocol

Coronary computed tomographic angiography was performed within 2 months (median interval of 6 days) before ICA using a dual-source CT scanner (Somatom Definition FLASH, Siemens Healthcare, Germany) or a 256-slice CT scanner (Revolution CT, General Electric, MA, USA). For the prospectively ECG-triggered CCTA, the patients with body mass index (BMI) < 24 kg/m^2^ were scanned at 100 kV, and those with BMIs of ≥ 24 kg/m^2^ were scanned at 120 kV. The tube current was regulated by automatic exposure control. The acquisition window was performed within the 70% R-R interval for heart rates (HR) of < 60 bpm, 40–70% R-R interval for HRs of 60–80 bpm, and 30–40% R-R interval for HRs of > 80 bpm. Bolus-tracking was conducted by placing the region of interest in the root of the aorta, and images were automatically acquired 6 s after a predefined threshold of 100 Hounsfield units (HU) was reached. The scanning range was set from the tracheal bifurcation to 1 cm below the diaphragm. The contrast agent was injected with a dual-head power injector (Stellant D, Medrad, PA, USA) through an 18–20-gauge intravenous needle placed in the right antecubital vein. Then, 50–70 ml of contrast agent (Ultravist, 370 mg iodine/ml, Bayer, Germany) were injected, followed by 30 ml of saline as a bolus chaser with an injection rate of 4.5–5 ml/s for all phases.

### CCTA Analysis

Image quality was assessed by the two experienced radiologists based on trans-axial images following the Society of CCT guidelines.

A separate commercial software with a semi-automated 3-dimensional contour detection algorithm (QAngio CT Research Edition version 3.1.4, MEDIS Medical Imaging Systems, Leiden, the Netherlands) was used to quantify CTO lesion metrics. Predefined fixed intensity cutoff values on the HU were selected to assess the plaque constitution. Currently, different cut-off values are available in the literature, which is obtained by comparing CTA with intravascular ultrasound virtual histology (IVUS VH) or histological examination. For the current analysis, the fixed HU cut-off values used for classifying were: dense calcium (>350 HU), fibrous (131–350 HU), fibro-fatty (76–130 HU), and necrotic core (−30 to 75 HU). These values were initially based on the paper by Brodoefel et al. (14) and empirically optimized using three representative training sets. The volume and proportion of each component were calculated.

Coronary tree lumen and wall were automatically extracted and manually corrected when necessary. All CTO characteristics were retrieved. Multiple occlusion was defined as at least two completely interrupted contrast media opacities with an interval of at least 5 mm. Stump morphology was categorized as tapered or blunt. Proximal side branch was defined as any side branch within 3 mm near the entrance. Bending was recognized as the presence of at least one bend of >45 degrees throughout the occlusion route. Severe calcium was defined as the presence of high-density plaque involving ≥50% of the vessel cross-sectional area (CSA). Additionally, occlusion length and proximal and distal vessel diameters were analyzed quantitatively. Occlusion length was then categorized as either <20 or ≥20 mm. CT-RECTOR, KCCT, and CTA J-CTO scores for each individual CTO were then calculated.

### Coronary Angiography and PCI Procedure Analysis

Coronary angiographic analyses were conducted by the two experienced cardiologists blinded to the results from clinical characteristics and CCTA. Angiographic variables, including target vessel, multiple occlusions, ostial CTO, blunt entry site, side branch, bending, calcification, occlusion length, bridging collaterals, the degree of retrograde collaterals according to the Rentrop classification, and severe proximal and distal vessel diseases, were determined as previously described. Procedural indexes were retrieved, including access site, guiding catheter size, retrograde approach, over-the-wire balloon support, GW numbers, and procedure outcomes. J-CTO and PROGRESS-CTO scores were then calculated according to previous studies.

### Statistical Analysis

Continuous variables were described as the mean and SD or median with interquartile range (IQR). The Student's *t*-test or the Mann–Whitney *U*-test was used to assess differences in continuous variables among groups. Categorical variables were expressed as absolute numbers and frequencies (%) and were compared with Pearson's chi-squared test or Fisher's exact test. As calcium and fibrous were both considered to be hard compositions, the receiver operating characteristic (ROC) curve of necrotic core percentage was generated, and the cutoff value was used to divide the patients into soft or hard CTO groups. Kaplan–Meier analysis was performed to compare outcomes between groups. A Log-rank test was adopted to compare rates of endpoints. To explore the risk factors associated with successful GW crossing of the CTO within 30 min, we performed multivariate regression using the overall, soft, and hard cohorts. Independent variables were selected to develop a prediction model for hard CTOs. The difficulty score for each hard CTO lesion was calculated by assigning points for each factor and then summing all points. The performance of the prediction model was assessed by the ROC curve. All analyses were performed using SPSS 21.0 (IBM Corp., Armonk, NY, USA). Differences obtained using a two-tailed test and *p* < 0.05 were deemed statistically significant.

## Results

### Clinical Characteristics

**Figure 1:**
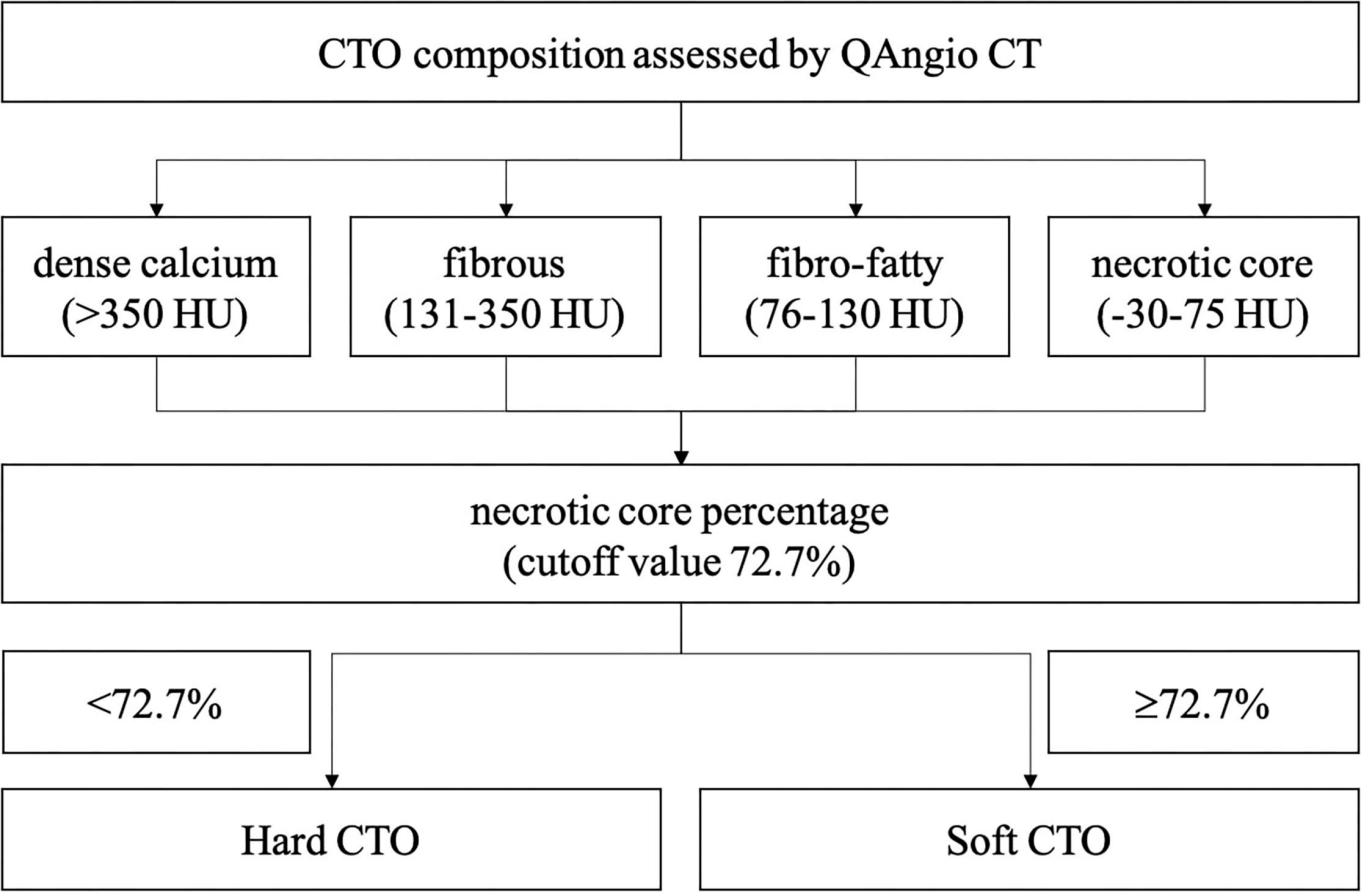
Chronic total occlusion **(CTO)** classification system.

A total of 201 patients (207 lesions) were enrolled. A ROC curve of necrotic core percentage was generated, and the cutoff value of 72.7% was used to divide the patients into soft or hard CTO groups. Lesions with the necrotic core proportion of ≥72.7% of the entire CTO plaque were defined as soft CTO (*n* = 42), whereas those with < 72.7% were defined as hard CTO (*n* = 165). Figure 1 shows how the CTO classification system works. No differences in baseline clinical characteristics, including age, sex, smoking history, and medical histories, were observed (Table 1). Most of the patients were attempted for the first time PCI (86.5%).

**Table 1:** Baseline clinical characteristics.

	**Overall (*n* = 207)**	**Soft CTO (*n* = 42)**	**Hard CTO (*n* = 165)**	***p*-Value**
Age, years	58.7 ± 10.7	57.5 ± 12.1	59.0 ± 10.3	0.295
Male, *n* (%)	176 (85.0)	39 (92.9)	137 (83.0)	0.111
BMI, kg/m^2^	26.3 ± 3.3	26.5 ± 3.5	26.3 ± 3.3	0.759
Smoking history, *n* (%)	112 (54.1)	24 (57.1)	88 (53.3)	0.658
Hypertension, *n* (%)	131 (63.3)	24 (57.1)	107 (64.8)	0.355
Diabetes, *n* (%)	62 (30.0)	13 (31.0)	49 (29.7)	0.874
Hyperlipidemia, *n* (%)	82 (39.6)	14 (33.3)	68 (41.2)	0.351
Previous MI, *n* (%)	53 (25.6)	12 (28.6)	41 (24.8)	0.622
Previous PCI, *n* (%)	39 (18.8)	6 (14.3)	33 (20.0)	0.398
Previous CABG, *n* (%)	2 (1.0)	0 (0)	2 (1.2)	1.000
Prior stroke, *n* (%)	19 (9.2)	5 (11.9)	14 (8.5)	0.699
Renal disease, *n* (%)	13 (6.3)	2 (4.8)	11 (6.7)	0.922
PVD, *n* (%)	10 (4.8)	2 (4.8)	8 (4.8)	1.000
LVEF (%)	61.7 ± 8.3	61.0 ± 5.8	61.8 ± 8.8	0.113
eGFR, mL/min/1.73 m^2^	94.1 ± 16.1	92.8 ± 19.6	94.4 ± 15.2	0.987
TG, mmol/L	1.8 ± 1.0	1.7 ± 0.9	1.8 ± 1.0	0.692
TC, mmol/L	3.9 ± 1.0	3.7 ± 1.0	3.9 ± 0.9	0.205
LDL, mmol/L	2.3 ± 0.8	2.1 ± 0.8	2.3 ± 0.8	0.312
HDL, mmol/L	1.0 ± 0.2	1.0 ± 0.2	1.0 ± 0.2	0.126
FBG, mmol/L	6.6 ± 2.3	6.5 ± 2.3	6.6 ± 2.3	0.393
HbA1c (%)	6.4 ± 1.1	6.4 ± 1.1	6.4 ± 1.1	0.487
Occlusion time ≥ 12 months or unknown, *n* (%)	137 (66.2)	31 (73.8)	106 (64.2)	0.242
Reattempt of failed CTO PCI, *n* (%)	28 (13.5)	4 (9.5)	24 (14.5)	0.396

*CTO, chronic total occlusion; BMI, body mass index; MI, myocardial infarction; PCI, percutaneous coronary intervention; CABG, coronary artery bypass grafting; PVD, peripheral vascular disease; LVEF, left ventricular ejection fraction; eGFR, estimated glomerular filtration rate; TG, triglyceride; TC, total cholesterol; LDL, low-density lipoprotein; HDL, high-density lipoprotein; FBG, fasting blood glucose*.

### Coronary Angiographic Characteristics

**Figure 2:**
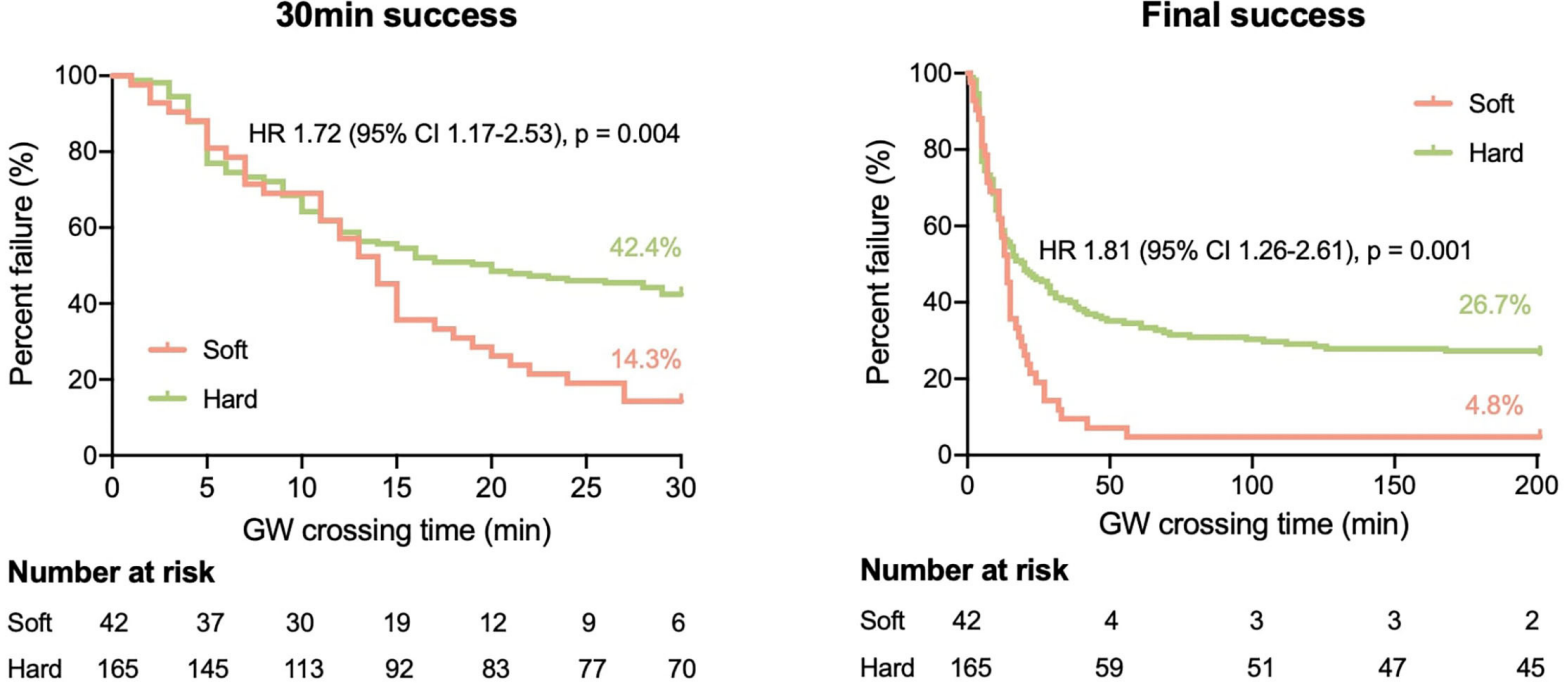
Kaplan–Meier curves of successful 30 min (left) and final (right) guidewire (GW) crossing in patients with soft and hard CTO.

**Figure 3:**
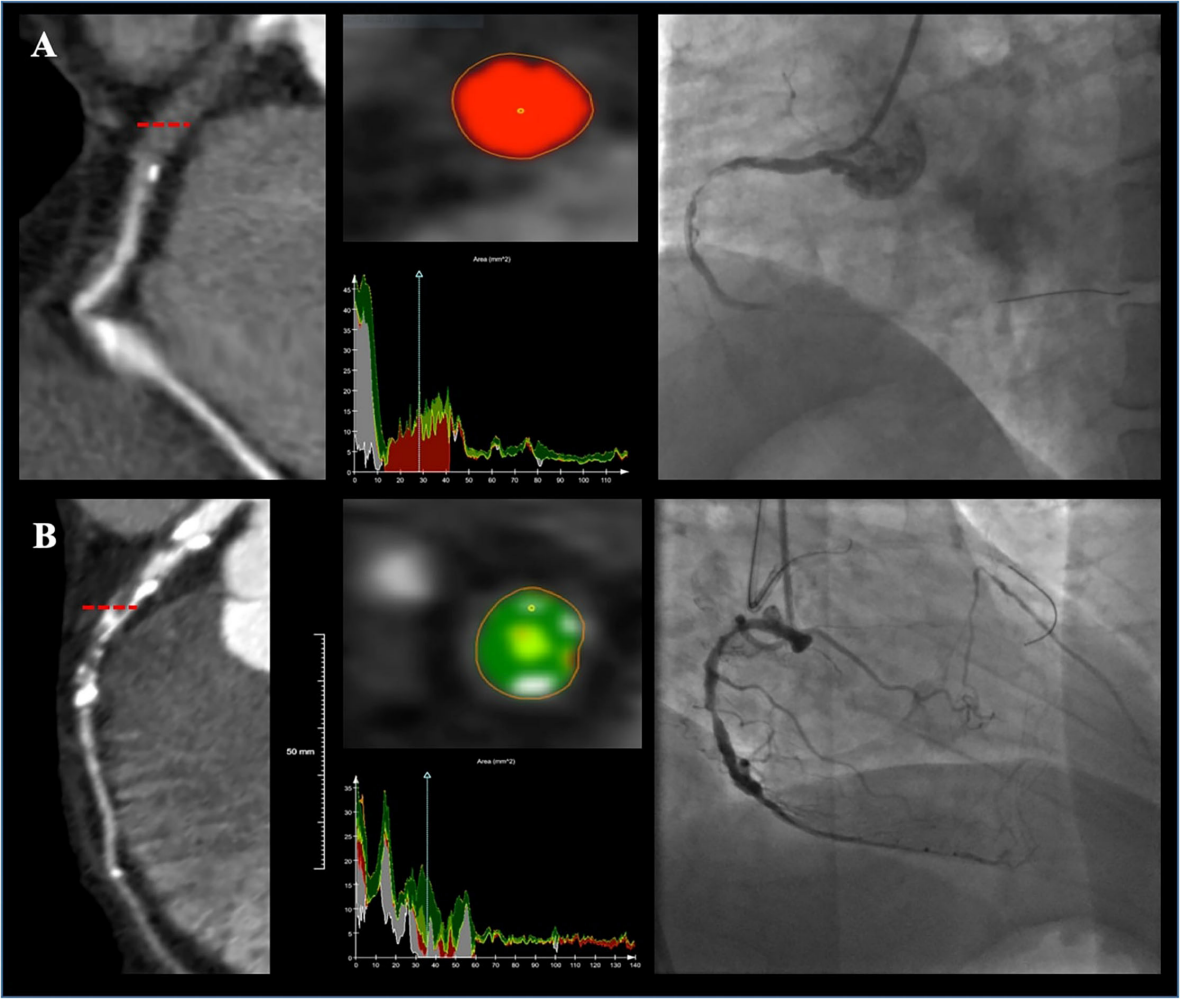
Two representative cases showing plaque compositions and guidewire (GW) crossing outcomes. Lesion **(A)** was a right coronary artery (RCA) lesion with soft CTO (80.7% necrotic core, 17.4% fibrous fatty, 1.6% fibrous, and 0% dense calcium) and the GW was successfully passed at 20 min. Lesion **(B)** was a left anterior descending artery (LAD) lesion with hard CTO (46.4% fibrous, 21.9% dense calcium, 17.8% fibrous fatty, and 13.5% necrotic core) and the GW was failed to be advanced cross the CTO.

The most common target vessel for both groups was the right coronary artery (RCA) (Table 2). No obvious difference in plaque characteristics was detected except for calcium (4.8 vs. 26.1%, *p* = 0.003). Only two (4.8%) soft CTOs had calcium. Patients with hard CTO underwent the retrograde wiring approach more frequently (11.5 vs. 0%, *p* = 0.045) and consumed more guiding wires (3.8 ± 2.0 vs. 3.0 ± 1.2, *p* = 0.016). However, the rate of successful GW crossing within 30 min (57.6 vs. 85.7%, *p* = 0.004) and final success (73.3 vs. 95.2%, *p* = 0.001) was much lower in the hard CTO group. The difference was observed beginning at about 13 min (Figure 2). Eventually, stents were implanted in 36 (85.7%) lesions of the soft CTO group and 116 (70.3%) of the hard CTO group. Figure 3 shows representative cases of plaque compositions and GW crossing outcomes.

**Table 2:** Angiographic and procedural characteristics.

	**Overall (*n* = 207)**	**Soft CTO (*n* = 42)**	**Hard CTO (*n* = 165)**	***p*-Value**
**Angiographic characteristics**
Target vessel				0.177
LM, *n* (%)	2 (1.0)	0 (0)	2 (1.2)	
LAD, *n* (%)	85 (41.1)	12 (28.6)	73 (44.2)	
LCX, *n* (%)	22 (10.6)	7 (16.7)	15 (9.1)	
RCA, *n* (%)	98 (47.3)	23 (54.8)	75 (45.5)	
Multiple occlusion, *n* (%)	9 (4.3)	2 (4.8)	7 (4.2)	1.000
Ostial location, *n* (%)	10 (4.8)	1 (2.4)	9 (5.5)	0.670
Blunt stump at entry, *n* (%)	76 (36.7)	12 (28.6)	64 (38.8)	0.220
Side branch at entry, *n* (%)	90 (43.5)	14 (33.3)	76 (46.1)	0.137
Bending > 45°, n (%)	48 (23.2)	11 (26.2)	37 (22.4)	0.606
Calcium, any, *n* (%)	45 (21.7)	2 (4.8)	43 (26.1)	0.003
Heavy calcium, *n* (%)	20 (9.7)	0 (0)	20 (12.1)	0.037
Occlusion length ≥ 20 mm, *n* (%)	85 (41.1)	16 (38.1)	69 (41.8)	0.661
Bridging collaterals, *n* (%)	21 (10.1)	5 (11.9)	16 (9.7)	0.891
Retrograde collaterals grade ≥ 2, *n* (%)	195 (94.2)	37 (88.1)	158 (95.8)	0.127
Severe proximal vessel disease, *n* (%)	68 (32.9)	16 (38.1)	52 (31.5)	0.418
Severe distal vessel disease, *n* (%)	86 (41.5)	16 (38.1)	70 (42.4)	0.611
**Procedural characteristics**
Radial access, *n* (%)	85 (41.3)	17 (40.5)	68 (41.5)	0.908
Guiding catheter				0.609
6-F, *n* (%)	136 (65.7)	29 (69.0)	107 (64.8)	
7-F, *n* (%)	71 (34.3)	13 (31.0)	58 (35.2)	
Retrograde injection, *n* (%)	97 (46.9)	18 (42.9)	79 (47.9)	0.560
Retrograde wiring approach, *n* (%)	19 (9.2)	0 (0)	19 (11.5)	0.045
Over-the-wire balloon support, *n* (%)	10 (4.8)	0 (0)	10 (6.1)	0.218
Simultaneous PCI for non-CTO lesion, *n* (%)	73 (35.3)	17 (40.5)	56 (33.9)	0.429
Mean number of wires, *n* (%)	3.6 ± 1.8	3.0 ± 1.2	3.8 ± 2.0	0.016
Successful GW crossing within 30, *n* (%)	131 (63.3)	36 (85.7)	95 (57.6)	0.001
Final successful GW crossing, *n* (%)	161 (77.8)	40 (95.2)	121 (73.3)	0.002
Successful implantation of stent, *n* (%)	152 (73.4)	36 (85.7)	116 (70.3)	0.044
Dissection or perforation, *n* (%)	9 (4.2)	2 (4.8)	7 (4.2)	1.000

*CTO, chronic total occlusion; LM, left main; LAD, left anterior descending; LCX, left circumflex; RCA, right coronary artery; PCI, percutaneous coronary intervention; GW, guidewire*.

### CCTA Characteristics

In terms of characteristics detected by CCTA, the proximal reference vessel diameter in lesions with hard CTO was much larger (3.0 ± 0.7 vs. 2.8 ± 0.7, *p* = 0.013) (Table 3). Hard CTO was more likely to characterized by blunt stump at the entry (39.4 vs. 21.4%, *p* = 0.030), proximal side branch (35.8 vs. 14.3%, *p* = 0.007), and calcification (*p* < 0.001).

**Table 3:** Computed tomographic characteristics.

	**Overall (*n* = 207)**	**Soft CTO (*n* = 42)**	**Hard CTO (*n* = 165)**	***p*-Value**
Occlusion length, mm	20.3 ± 15.8	16.3 ± 10.4	21.3 ± 16.8	0.066
Occlusion length ≥ 15 mm, *n* (%)	107 (51.7)	21 (50.0)	86 (52.1)	0.806
Occlusion length ≥ 20 mm, *n* (%)	80 (38.6)	12 (28.6)	68 (41.2)	0.133
Multiple occlusion, *n* (%)	10 (4.8)	1 (2.4)	9 (5.5)	0.670
Proximal reference vessel diameter, mm	3.0 ± 0.7	2.8 ± 0.7	3.0 ± 0.7	0.013
Distal reference vessel diameter, mm	2.2 ± 0.5	2.2 ± 0.5	2.2 ± 0.5	0.755
Blunt stump, *n* (%)	74 (35.7)	9 (21.4)	65 (39.4)	0.030
Side branch, *n* (%)	65 (31.4)	6 (14.3)	59 (35.8)	0.007
Bending > 45°, *n* (%)	42 (20.3)	9 (21.4)	33 (20.0)	0.837
Calcium, any, *n* (%)	80 (38.6)	0 (0)	80 (48.5)	<0.001
Calcium ≥ 50% CSA, *n* (%)	63 (30.4)	0 (0)	63 (38.2)	<0.001

*CTO, chronic total occlusion; CSA, cross-sectional area*.

### Risk Scoring Systems

**Figure 4:**
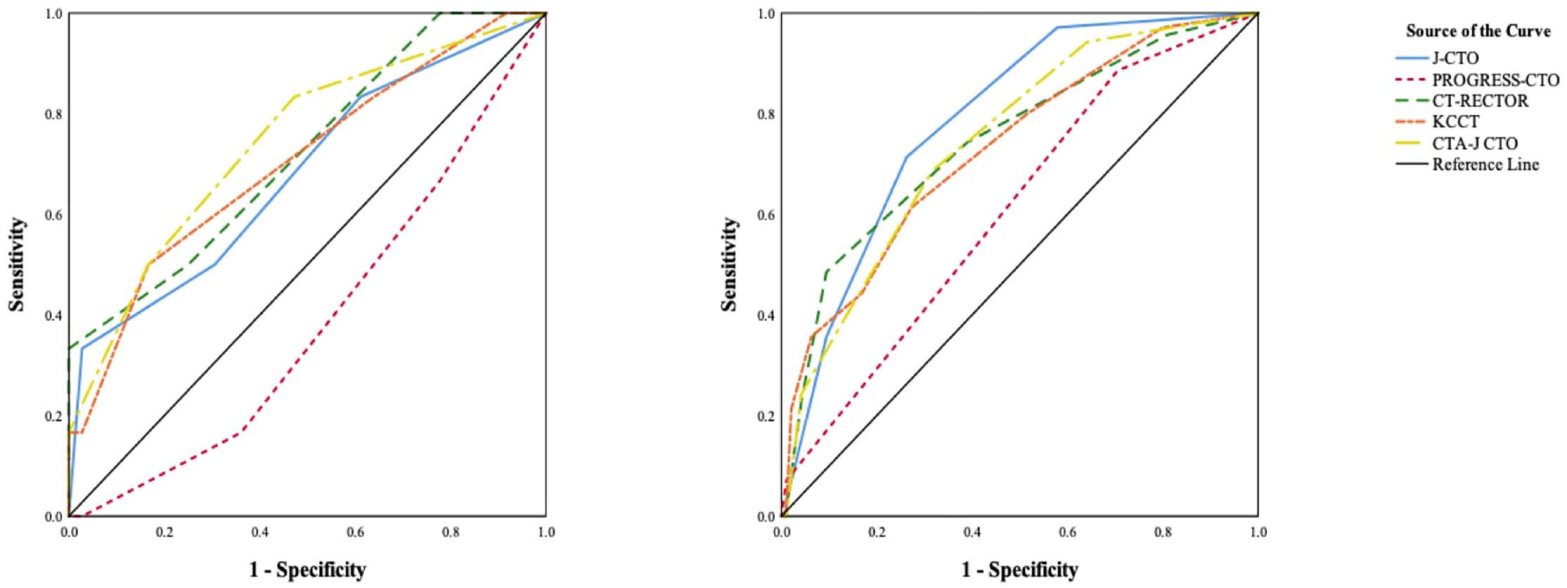
Receiver operating characteristics (ROC) curves of previous scoring systems in patients with soft CTO (left) and hard CTO (right).

We compared the current predictive scores between the soft CTO and hard CTO groups (Table 4). The J-CTO, KCCT, and PROGRESS-CTO scores were similar between the two groups. CT-RECTOR (1.8 ± 1.2 vs. 1.2 ± 0.9, *p* = 0.002) and CTA-J CTO (1.6 ± 1.3 vs. 0.8 ± 0.9, *p* < 0.001) were much higher in the hard CTO group. The predictive values of each scoring system, as evaluated by ROC curves, are shown in Figure 4.

**Table 4:** Predictive assessment of different scoring systems.

	**Overall (*n* = 207)**	**Soft CTO (*n* = 42)**	**Hard CTO (*n* = 165)**	***p*-Value**
J-CTO	1.4 ± 1.1	1.1 ± 1.0	1.4 ± 1.1	0.057
PROGRESS-CTO	1.2 ± 0.8	1.1 ± 0.8	1.2 ± 0.8	0.671
CT-RECTOR	1.7 ± 1.2	1.2 ± 0.9	1.8 ± 1.2	0.002
KCCT	2.4 ± 1.8	1.9 ± 1.1	2.5 ± 1.9	0.162
CTA-J CTO	1.5 ± 1.3	0.8 ± 0.9	1.6 ± 1.3	<0.001

### Independent Predictive Factors

To investigate the independent predictors of successful 30 min GW crossing, we performed the multivariate analysis that included clinical and CCTA variables, such as age, sex, BMI, previous failed attempt, blunt stump, side branch, bending, and calcification.

For patients with hard CTO, conventional predictive factors established in the J-CTO, PROGRESS-CTO, CT-RECTOR, KCCT, and CTA-J CTO scoring systems, including previous failed attempt [hazard ratio (*HR*) 0.215, 95% *CI* 0.070–0.664, *p* = 0.008], proximal side branch (*HR* 0.250, 95% *CI* 0.110–0.571, *p* = 0.001), bending > 45 degrees (*HR* 0.066, 95% *CI* 0.020–0.218, *p* < 0.011), and calcium ≥ 50% cross-sectional area (*HR* 0.325, 95% *CI* 0.147–0.717, *p* = 0.005) were validated. Additionally, distal reference diameter ≤ 2.5 mm was proved to be associated with GW failure (*HR* 4.748, 95% *CI* 1.698–13.275, *p* = 0.003). For patients with soft CTO, only blunt entry was proved to be an independent predictive factor of GW failure within 30 min (*HR* 0.081, 95% *CI* 0.012–0.563, *p* = 0.011) (Table 5).

**Table 5:** Independent predictors of 30 min GW crossing CTO.

**Variables**	**Overall**			**Soft CTO**			**Hard CTO**		
	**HR**	**95%CI**	***P*-Value**	**HR**	**95%CI**	***P*-Value**	**HR**	**95%CI**	***P*-Value**
Blunt entry	0.444	0.216–0.913	0.027	0.081	0.012–0.563	0.011	…	…	…
Failed attempt	0.203	0.077–0.536	0.001	…	…	…	0.215	0.070–0.664	0.008
Distal reference diameter^*^			…	…	…	…	4.748	1.698–13.275	0.003
Proximal side branch	0.352	0.166–0.748	0.007	…	…	…	0.250	0.110–0.571	0.001
Bending > 45°	0.232	0.100–0.536	0.001	…	…	…	0.066	0.020–0.218	<0.001
Calcium ≥ 50% CSA	0.303	0.149–0.616	0.001	…	…	…	0.325	0.147–0.717	0.005

*Data were analyzed using a Cox regression model*.

* *Distal reference diameter was divided into ≤2.5 and >2.5 mm. CTO, chronic total occlusion; HR, hazard ratio; CI, confidence interval; CSA, cross-sectional area*.

### Development of the HCTO Score

**Figure 5:**
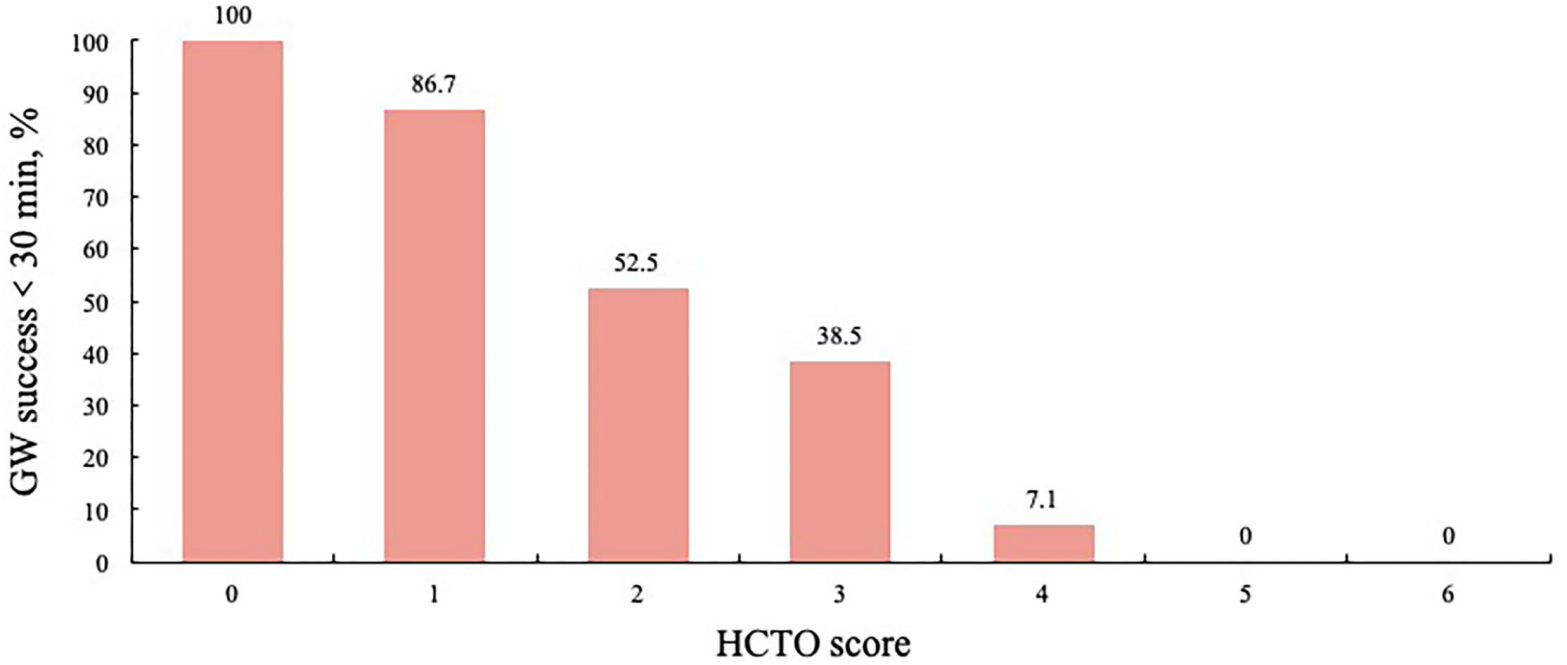
Relationship between HCTO score and GW crossing within 30 min.

Each independent variable developed in the regression analysis was assigned an integer score. According to the beta coefficients, previous failed attempts (1.536), distal reference diameter ≤2.5 mm (1.558), proximal side branch (1.386), and calcium ≥ 50% cross-sectional area (1.124) were assigned an integer score of 1. Bending > 45 degrees (2.720) was assigned 2. For each hard CTO lesion, a total difficulty score for PCI, which is the HCTO score, was determined by summing all points. The area under the ROC curve for the HCTO score was 0.802. Figure 5 shows the relationship between the HCTO scores and successful GW crossing within 30 min. Then, the patients were categorized into 3 groups with dramatically increase in difficulty: easy (HCTO score of 0–1), intermediate (HCTO score of 2–3), and hard (HCTO score of 4–6). The success rate of CTO recanalization within 30 min for each group was 89.2, 53.5, and 5.6%, respectively.

## Discussion

In this study, we innovatively divided CTO lesions into soft and hard ones based on pathological considerations, rather than calcification and non-calcification and further evaluated the impact of plaque composition on the successful GW crossing within 30 min of CTO lesions. Our study revealed that the outcomes and predictive factors in soft CTO that mainly comprised a necrotic core and hard CTO that mainly consisted of fibrous and/or dense calcium are significantly different. The majority lesions with soft CTO could be successfully recanalized, and only proximal entry morphology had an effect on the outcome. The success rate of hard CTO PCI was much lower, which was influenced by several conventional clinical and plaque characteristics. Previous studies usually classify plaques according to the degree of calcification, ignoring the other unfavorable factor for the success, fibrous. In this study, we comprehensively evaluated the compositions of CTO lesions and further established a simpler and more satisfactory classification tool. This is the main innovation of our research.

During the development of CTO therapy and experience accumulation process, numerous scoring systems have been established to predict the successful recanalization of CTO lesions, including J-CTO, CT-RECTOR, CL, PROGRESS-CTO, ORA, KCCT, and CTA-J CTO. Establishing scoring systems could effectively predict the success of CTO recanalization, accurately select appropriate patients for attempting PCI, and ultimately achieve a satisfactory immediate and long-term prognosis. However, these scoring systems exhibited moderate-level performance in predicting the technical outcome of CTO PCI (15) and experienced in our center.

A thorough understanding of CTO pathophysiology is critical to explain the phenomenon, optimize predictive models, and further develop newer techniques and technologies. In essence, most CTOs are caused by soft plaque rupture followed by thrombotic coronary occlusion and organization of thrombotic substances, while a minority of CTOs are caused by the progression of atheroma (16). Except for proximal and distal fibrous caps, the occluded segment materials were biologically active with inflammation, neovascularization, and recanalization, giving rise to different CTO compositions. Similar to non-CTO lesions in patients with stable angina, the composition of CTO lesions mainly consists of three types, namely, necrotic core, fibrous, and dense calcium (17). Previous studies exploring GW crossing predictors always focus on the severity of calcification in terms of plaque characteristics, which revealed that calcification is likely to be a predictive factor of failed GW crossing within 30 min. However, these investigations have underestimated the effect of low-density components on the success of CTO PCI, including the other unfavorable factor for the success, fibrous. This may be attributable to the limited ability of CCTA to evaluate.

Intravascular ultrasound (IVUS) is the gold standard for the diagnosis of plaque characteristics, but it is limited due to its high cost and invasive nature (18). Most importantly, IVUS could not be applied in CTO lesions with failed GW crossing. CCTA is a robust non-invasive method to analyze the presence, extent, and severity of coronary atherosclerosis with high accuracy (19, 20). Previous scoring systems, including CT-RECTOR, KCCT, and CTA-J CTO, established grading rules of CTO difficulty using CCTA indexes and provided a more accurate non-invasive tool for predicting the time-efficient GW passaging and final procedure success (21, 22). In our study, J-CTO and PROGRESS-CTO scores were similar between soft CTO and hard CTO groups, whereas CT-RECTOR and CTA-J CTO scores were much higher in the hard CTOs, indicating that CCTA derived scoring systems are more likely to distinguish lesion characteristics, including plaque compositions, compared with ICA derived parameters.

Recent advances in CCTA technology have enabled us to better understand the pathophysiology of coronary atherosclerosis and its association with future CVD risk by providing additional coronary atherosclerosis information, such as plaque characteristics and volumes (23, 24). In our study, non-invasive plaque compositions were analyzed based on the CCTA data using the semi-automated plaque analysis software, which has been used in previous studies (25, 26). We divided the patients into soft and hard CTO groups according to the percentage of the necrotic core. Thus, patients with hard CTO were mainly composed of fibrous and/or calcified plaques. This grouping method fully considered the impact of low-density non-calcified plaques.

According to our analysis, conventional predictive factors, including previous failed attempts, proximal side branch, bending, calcification, and distal reference diameter were confirmed in patients with hard CTO. It is worth mentioning that our study complemented previous observations by introducing a novel predictor distal reference diameter. Yamamoto et al. (27) showed that CTO lesions with positive remodeling apparently have unstable plaques, such as lipid-rich plaque, macrophages, and underlying plaque morphology of plaque rupture or attenuated plaque. This explained why greater distal vessel diameter is associated with an increased GW passage. Even though a blunt stump has been frequently reported as a significant prognostic factor for PCI failure in previous angiographic research, it was not an independent predictor of GW outcome in the hard CTO group. Interestingly, for patients with soft CTO, only entry morphology was proved to be a predictive factor of GW failure within 30 min. The blunt entry was proved to be the single risk factor of failed GW 30 min success. Other characteristics of the CTO segment are irrelevant.

Our study provided a novel approach for the preoperative evaluation of CTO PCI. Based on the above results, further evaluation of CTO lesions should be altered by first analyzing plaque compositions using non-invasive CCTA. Complete assessment of CTO lesion by adding plaque composition analysis to the conventional predictive factors would optimize the treatment strategies of CTO lesions.

Grouping CTO lesions according to the proportion of necrotic core is reasonable and necessary in predicting the GW crossing within 30 min. A soft CTO with a necrotic core is more likely to be recanalized compared with a hard CTO with fibrous and/or dense calcium. Different plaque types have varying predictive factors.

Several limitations of this study need to be addressed. First, the retrospective nature of our study may potentially result in biases. Second, the sample size is small because CCTA was not routinely performed in patients with CTO. Third, we should keep in mind that most of the lesions used an antegrade crossing approach, so the results are more suitable for a certain patient population. Therefore, the findings of our study should be considered as hypothesis-generating, with further large-scale studies warranted to confirm our findings. In addition, this classification method is mainly based on lesion composition recognition, which cannot be realized by the naked eye. This disadvantage might limit its clinical application.

## Data Availability Statement

The raw data supporting the conclusions of this article will be made available by the authors, without undue reservation.

## Ethics Statement

The studies involving human participants were reviewed and approved by Beijing Anzhen Hospital Ethics Committee. Written informed consent for participation was not required for this study in accordance with the national legislation and the institutional requirements.

## Author Contributions

XS and YH contributed substantially to the design of the present study. DZ performed the statistical analyses and drafted the manuscript. All co-authors participated in the interpretation of data and critically revised the manuscript. All authors have approved the final version of the manuscript.

## Funding

This work was supported by the Capital Health Research and Development of Special of China (Grant Number: 2018-2-2063) and the National Natural Science Foundation of China (Grant Number: 81971569).

## Conflict of Interest

The authors declare that the research was conducted in the absence of any commercial or financial relationships that could be construed as a potential conflict of interest.

## Publisher's Note

All claims expressed in this article are solely those of the authors and do not necessarily represent those of their affiliated organizations, or those of the publisher, the editors and the reviewers. Any product that may be evaluated in this article, or claim that may be made by its manufacturer, is not guaranteed or endorsed by the publisher.
